# Emerging Cytotoxic Alkaloids in the Battle against Cancer: Overview of Molecular Mechanisms

**DOI:** 10.3390/molecules22020250

**Published:** 2017-02-08

**Authors:** Zeina Habli, Georgio Toumieh, Maamoun Fatfat, Omar Nasser Rahal, Hala Gali-Muhtasib

**Affiliations:** 1 Department of Biology and Department of Anatomy, Cell Biology and Physiological Sciences, American University of Beirut, Beirut 1107 2020, Lebanon; zeina1hb@gmail.com (Z.H.); gjt01@mail.aub.edu (G.T.); mf53@aub.edu.lb (M.F.); 2 School of Medicine, Saba University School of Medicine, Saba, Dutch Caribbean 5016121, The Netherlands; omar2.rahal@gmail.com

**Keywords:** alkaloids, apoptosis, cancer therapeutics, medicinal plants, molecular activity

## Abstract

Considered as the second deadliest disease globally, cancer has captured the attention of researchers who have been trying with perseverance to decode its hidden aspects, to find new prognosis methods, and to develop better and more effective treatments. Plants have continuously offered an excess of unique secondary metabolites with remarkable biological applications. Alkaloids, one of the most abundant metabolites, constitute a large conglomerate of basic heterocyclic nitrogen-containing natural compounds which are normally produced by plants as toxic substances. Out of the 27,000 different alkaloids, more than 17,000 have displayed diversified pharmacological properties including anticancer activities. These metabolites have been classified either according to their chemical structures or their taxonomic origin. None of the researched alkaloids have been classified according to their molecular mechanism of action against cancer. In fact, only a fraction of the tremendous number of anticancer alkaloids has been copiously mentioned in journals. Here, we aim to provide a summary of the literature on some of the promising anticancer alkaloids that have not been well discussed previously and to classify them according to their molecular mechanisms of action. This review will provide a better understanding of the anticancer mechanisms of these promising natural products that are a rich reservoir for drug discovery.

## 1. Introduction

Although neither contagious nor infectious, cancer roars as the second leading cause of death worldwide, grieving an estimated 8.2 million deaths in 2012 according to the World Health Organization (WHO data, 2012). Systematic research in the cancer field has reported that the rapid uncontrollable multiplication and spreading of abnormal cells is the foremost cause of cancer development and progression. In principle, dynamic abnormalities constantly emerge in the genome of cancer cells during cellular division, and the accumulation of such genetic mutations, mainly those targeting tumor suppressor proteins, growth factors and transcription factors, govern the transformation of normal cells into malignant cancers. These hostile cells have the ability to evade apoptosis, avoid immunity detection, and replicate limitlessly [1,2]. Although huge efforts have been invested in the development of a cure for this fatal disease, the number of cancer patients has been increasing dramatically with millions of new cases arising each year [3]. As a matter of fact, experts have anticipated that the annual cancer cases will increase from 14 million in 2012 to 22 million within the next two decades (WHO data, 2012). Furthermore, the disseminated resistance of cancer cells to apoptosis and to effective chemo-therapeutic agents creates a great threat and urges the discovery of novel adjuvant therapies [4].

There is no doubt that medicinal plants, the oldest form of medicaments employed by man in the battle against the majority of diseases and disorders [5], could serve as a safe alternative substitute for many of the synthetic drugs used in current clinical therapies [6]. In fact, herbal medicine constitutes the largest and most valuable source of natural agents that are proven to have potent effects against many varieties of maladies, including cancer [7]. Ample evidence confirms that the biological activity of these plants stems mainly from their secondary metabolites, which include the promising alkaloids. Interestingly, this family of compounds has recently gained great interest in the cancer treatment province due to their anti-proliferative and anti-angiogenic effects [8].

The majority of studies focus on the induced cytotoxicity of well-known alkaloids such as vinblastine, topotecan, taxol, vincristine and vinflunine that are used clinically in cancer therapy worldwide [9]. Screening for novel agents has led to the discovery of new alkaloids that showed promising anti-neoplastic and apoptotic abilities in several cancer cell lines. In this paper, we intend to shed light on promising emerging alkaloids that could be potentially translated into the clinical setting as cancer therapeutics. In fact, we seek to integrate the extensive knowledge and wide literature on anticancer natural agents with the purpose of classifying 20 new alkaloids in a novel way by principally investigating and summarizing their molecular mechanism of action against cancer.

## 2. Plant-Derived Alkaloids: *A Fountain of Bioactive Agents*

Since the emergence of herbal medicines, alkaloids have gained ascending popularity due to their incontestable physiological properties [10]. From an evolutionary perspective, these secondary metabolites were employed by various plants for protection against herbivorous predators [11]. Then, they were soon exploited by antiquity hunters and transformed into the venom used in poisonous arrows [12]. Afterwards, at the dawn of the 19th century, studies were initiated on these important substances, and year after year, their biological activity began to emerge and get recognized in the medical field, making them one of the major components of several anticancer drugs [13,14].

Additionally, among the hundreds of screened natural secondary metabolites, alkaloids embody the largest group of low molecular weight organic nitrogenous compounds isolated from terrestrial and marine sources [14]. The structural diversity of this family is actually due to the wide number of amino acids used as building blocks. Indeed, the peptide ring that they contain has one or more of its hydrogen atoms replaced with various alkyl radicals, most of which contain oxygen. As a consequence, alkaloids are capable of interacting with a wide spectrum of molecules [10,15]. In fact, out of thousands of different alkaloids, about 17,000 molecules have displayed pronounced biological and pharmacological activities [16]. With their unlimited supply of endless structures as well as their relatively low toxicity and well-documented stability, alkaloids have been a base for the extraction and synthesis of hundreds of medications which are used to combat various ailments [14]. Consequently, alkaloids have undoubtedly revolutionized medicine as anti-inflammatory, antibacterial, and most importantly antitumor agents [10].

Studies have proven the efficacy of plant-derived alkaloids in oncogenesis suppression. Alkaloids are capable of modulating key signaling pathways involved in proliferation, cell cycle, and metastasis, making them the chief components of several clinical anticancer agents [17]. Interestingly, the molecular mechanisms that these secondary metabolites act on are numerous and effective in various ways against cancer cells. For instance, paclitaxel is one of the major agents used clinically against breast cancer, ovarian cancer, non-small cell lung cancer, and prostate cancer [18]. On the other hand, vincristine has shown to be highly effective in the treatment of lymphoid cancers such as acute lymphoblastic leukemia [19]. Most of these medically exploited alkaloids function as therapeutics by mainly provoking DNA damage, inducing apoptosis, and acting as anti-proliferative agents; however, their associated toxicity urges the finding of new natural compounds that have the potential to selectively target cancerous cells [20,21,22].

## 3. Emerging Cytotoxic Alkaloids: *Apoptotic Strategies*

Apoptosis plays many important roles in the development and survival of multicellular organisms. It is a well-executed operation that involves ordered morphological and biochemical events that precede cell death; these include DNA breakage/laddering, cell shrinkage, nuclear condensation, membrane blebbing (membrane-bound apoptotic bodies) and modulation of precise signaling circuitry. Hence, apoptosis represents an essential part of the normal cell cycle and a natural barrier to cancer development [23,24].

Any irreversible disturbance in the upstream regulators or downstream effector components of the apoptotic machinery leads to tumorigenesis. In fact, evading apoptosis and resisting cell death is one of the ten hallmarks of cancer which directs the process of cell immortalization. Nevertheless, any molecule that is able to induce and activate the apoptotic signaling proteins can be considered as a promising remedy for cancer treatment [1].

Due to their complex and diversified structures and activities, the conventional classification of alkaloids has been a challenge. Newly emerging plant-derived alkaloids have been found to exhibit cytotoxic effects on several cancer cells with promising IC_50_ values (Table 1). Below, we present a novel grouping scheme in the categorization of alkaloids, relying on their molecular mechanism of action as agents that induce apoptosis in cancerous cells.

### 3.1. DNA Damaging Alkaloids: A Useful Damage

Cells have evolved two strategies to fix their most vulnerable material, the DNA. The first is instant damaged-DNA repairing and the second is inactivating cells harboring damaged genomes. In fact, both steps are crucial to maintain the cellular genomic stability because non-repaired DNA damage is often coupled with genetic mutations, which in turn could lead to malignant transformation [1,50]. The surveillance machinery, commonly known as checkpoints, is able to halt cell cycle progression until the damage is repaired, but if the damage is irreversible, the prominent route would be apoptosis [51]. Cancer cells have developed the ability to surpass these guardian checkpoints and continue their division normally with the least interest of fixing their genetic injury [52]. In addition, PI3k/Akt signal transduction cascade is one of the several cellular proliferative pathways which promotes a normal cell cycle progression by modulating cyclins and pro-apoptotic proteins. Hence, overexpressed Akt leads to abnormal proliferative and anti-apoptotic signals that initiate the transformation of malignant tumors. Accordingly, many cytotoxic agents target DNA and the Akt pathway either directly or indirectly to block cell proliferation and induce apoptosis [53]. As such, alkaloids that promote apoptosis via inducing DNA damage seem to be a great option for cancer treatment.

**Figure 1:**
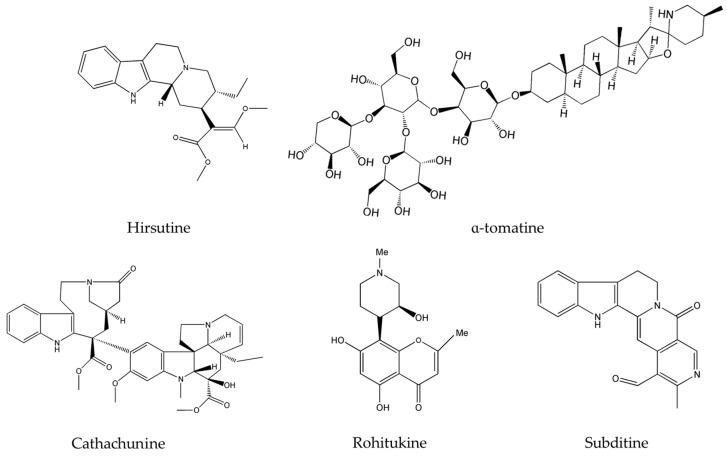
Chemical structures of hirsutine, α-tomatine, cathachunine, rohitukine and subditine.

Hirsutine (Figure 1), a major alkaloid extracted from plants of the genus *Uncaria*, has been reported to selectively inhibit Akt in human breast cancer cells, namely HER2 positive, p53-mutated MDA-MB-453, and BT474 cell lines [43]. Hirsutine was found to induce DNA damage as manifested by the up-regulation of γH2AX, a marker of DNA breakage [52], and to increase the expression of p-p38 MAPK. As a matter of fact, triple negative breast cancer (TNBC) represents an aggressive subtype of breast cancer responsible for disease recurrence, metastasis, and chemo-resistance [54]. Preliminary results of the anticancer in vitro efficacy of hirsutine showed that this alkaloid alone is capable of modulating the survival pathways and causing DNA damage, making it worthy to be incorporated with other drugs for the clinical treatment of TNBC [43]. However, hirsutine’s molecular activity has been only tested on breast cancer cells. Hence, further studies on this new metabolite could potentially unveil additional effects on more diverse types of cancers and lead to its development into an effective anticancer drug. On the other hand, α-tomatine (Figure 1) a glycoalkaloid common in *Lycopersicon esculentum* Mill., was found to inhibit Akt phosphorylation and suppress the extracellular signal-regulated kinase 1 and 2 (ERK1/2) without affecting the p38 MAPK [48].

Importantly, neoplastic development in the majority of cancers is promoted by many factors including an elevated level of the DNA-damaging reactive oxygen species (ROS). Normally, once the quantity of such species exceeds a certain limit, they are destroyed by anti-oxidant proteins; a balance of ROS radical species in the cell is delicately controlled and is vital for its well-functioning. Several anticancer drugs are known to disrupt the ROS balance in the cell favoring its abnormal increase and eventually leading to ROS-induced apoptosis. These agents mainly prevent early events in tumorigenesis, where ROS plays a central part [55]. In fact, cathachunine (isolated from *Catharanthus roseus* (L.) G.Don. commonly known as the Madagascar periwinkle) and subditine (extracted from *Nauclea subdita* (Korth.) Steud.), both presented in Figure 1, are two emerging alkaloids that act by increasing the intracellular levels of ROS in abnormal cells and are effective against leukemia [35] and prostate cancer [39]. Similarly, rohitukine (isolated from *Dysoxylum binectariferum* Hook.f.), had significant cytotoxicity against breast cancer, ovarian cancer, and lung cancer *via* ROS generation [41]. Topotecan, a commonly used chemotherapeutic agent and topoisomerase inhibitor, acts against many cancers, mainly cervical cancer and small cell lung cancer, by creating double-stranded DNA damage [56]. However, this alkaloid causes numerous toxic responses ranging from diarrhea, nausea, vomiting and fatigue to granulocytopenia, severe neutropenia, febrile neutropenia, severe thrombocytopenia, severe anemia, asthenia, and total alopecia [57,58,59]. The newly emerging alkaloids cathachunine, subditine and rohitukine cause DNA damage, as summarized in Table 2, and thus could form equally effective or even better drugs than topotecan, with less toxic responses and side-effects.

### 3.2. Apoptotic Alkaloids: Caspase Activators

Cysteine Asparatic Proteases, known as “Caspases”, specifically cleave their substrate proteins at the aspartate residues during apoptosis in a self-amplifying cascade. Caspases are important agents in the programmed cell death pathways. Two major pathways of caspase activation have been characterized, the intrinsic and the extrinsic pathways. They integrate the various apoptotic signals activated by DNA damage, cytotoxic agents, ROS production, aberrant oncogene expression and p53 activation. Once any of these signals is detected, the apoptotic machinery is activated [60,61].

**Figure 2:**
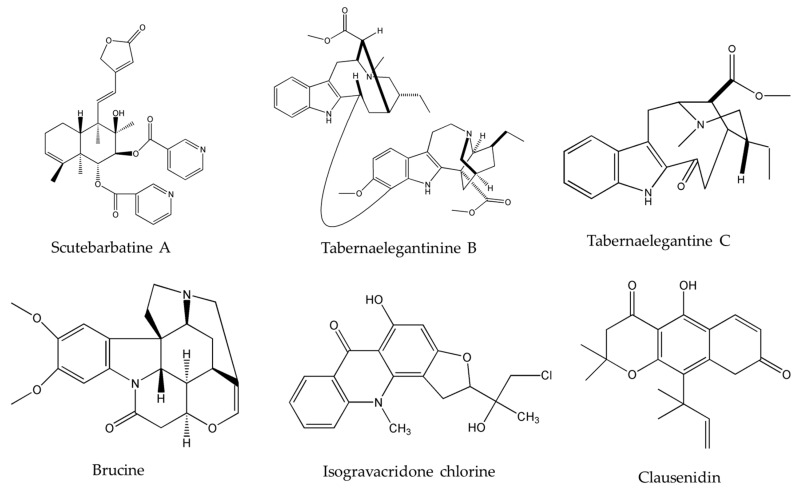

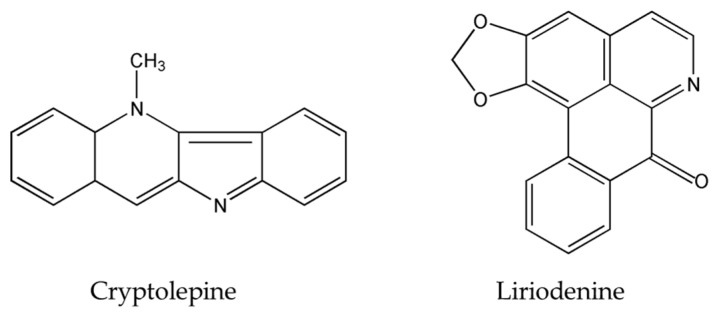
Chemical structures of scutebarbatine A, tabernaelegantinine B, tabernaelegantine C, brucine, isogravacridone chlorine, clausenidin, cryptolepine and liriodenine.

Upon investigating the molecular mechanism of apoptosis induction, subditine (Figure 1), a monoterpenoid indole alkaloid, isolated from the bark of *N. subdita*, was found to activate apoptosis in a dose-dependent fashion in human prostate cancer cells through the intrinsic pathway [39]. Subditine treatment led to higher p53 expression and down-regulation of Bcl2 and Bcl-xL thus inhibiting their anti-apoptotic activity. In addition, scutebarbatine A (Figure 2), a major alkaloid in *Scutellaria barbata* D.Don., was found to exhibit its anti-proliferative activity against human lung carcinoma cells, in a dose-dependent manner, through the cleavage of caspases-3 and -9 as well as the down-regulation of Bcl-2 protein expression which was confirmed by cytochrome c efflux to the cytosol [40]. Furthermore, tabernaelegantine C and tabernaelegantinine B (Figure 2), two alkaloids isolated from *Tabernaemontana elegans* Stapf, a medicinal plant, were found to activate caspase-8 in colon cells [42]. Additional apoptotic alkaloids, which were shown to be promising against a wide array of cancer cells, are demonstrated in Table 3 and chemical structures are in Figure 1 and Figure 2.

Primarily, anticancer therapies function via the modulation of several signaling pathways which ultimately result in the activation of caspases; the latter, in turn, executes and controls the apoptotic machinery to direct cancer cells towards cellular death [62]. Newly emerging alkaloids, derived from plants, activate an enormous set of caspases, as shown in Table 3, and have very acceptable IC_50_ values making them worthy of further testing. In fact, acquired inhibition of caspase activation is a major factor in chemo-resistance [63], thus combining these alkaloids with standard chemotherapy targeting multidrug resistant (MDR) cancer cells may result in significant inhibitory and apoptotic effects. 

### 3.3. Anti-Proliferative Alkaloids: Cell Growth Inhibitors

#### 3.3.1. Cell-Cycle Arrest

DNA replication and cellular division are processes which depend on the orchestration of various cascades of protein phosphorylation and on numerous checkpoints which supervise these two critical events and eventually lead to cell cycle completion. Cell cycle deregulation is one of the ten hallmarks of cancer transformation, and targeting one of its key modulators, which include cyclins, cdks (cyclin-dependent kinases), and tumor suppressor proteins (p53 and Rb), is a crucial step in arresting cell cycle and consequently inducing cancer cell death [64,65].

**Figure 3:**
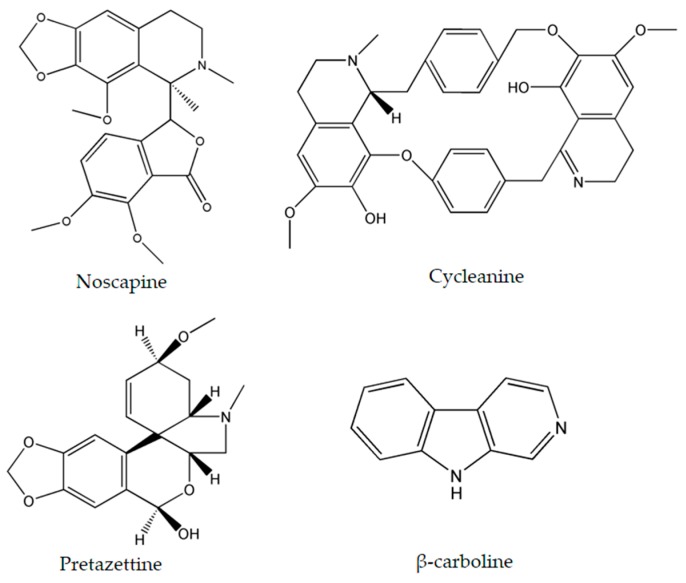
Chemical structures of noscapine, cycleanine, pretazettine and β-carboline.

The importance of several of the emerging alkaloids resides in their ability to act on cell cycle checkpoints to induce cell cycle arrest. This allows for repairs to be made, or in extreme cases directs the cell towards apoptosis [66]. For instance, noscapine (Figure 3) isolated from the opium flower *Papaver somniferum* L. induced G2/M arrest in various types of cancers such as breast cancer [28], lung cancer, and colorectal cancer [27]. Other alkaloids caused a halt in the S phase progression; for example, liriodenine (Figure 2) extracted from *Enicosanthellum pulchrum* (King) Heusden and isogravacridone chlorine isolated from the rue *Ruta graveolens* L. inhibited the proliferation of ovarian cancer [25] and breast cancer [33], respectively. Additionally, clausenidin (Figure 2), obtained from the shrub *Clausena excavate* Burum.f. induced cell cycle arrest of colon cancer at the G0/G1 phase [32]. Similarly, cycleanine (Figure 3) extracted from *Triclisia subcordata* Oliv., exerted the same effect as clausenidin on the cell cycle progression and induced G0/G1 immobilization of ovarian cancer cells [34]. Cryptolepine (Figure 2) extracted from several plants like the *Cryptolepis sanguinolenta*, the common wireweed *Sida acuta* Burm.f. and the perennial shrub *Sida cordifolia* L., caused G1/S as well as G2/M arrest in numerous cancers like lung adenocarcinoma [29], osteosarcoma [30], T-cell leukemia, multiple myeloma, histiocytic lymphoma, small cell lung cancer, renal adenocarcinoma, and cervical adenocarcinoma [31]. Last but not least, brucine (Figure 2), an effective alkaloid derived from the seeds of *Strychnos nux-vomica* L., inhibited the proliferation of human colon and lung cancer cells by arresting cell cycle at the G0/G1 phase [36,37]. Many of the FDA approved anticancer alkaloids function through cell cycle arrest [67]. For instance, taxol/paclitaxel, a diterpene alkaloid [10], extracted from *Taxus brevifolia* Nutt. and approved for the treatment of ovarian, breast and lung cancer, as well as Kaposi’s sarcoma, causes cell cycle arrest at the spindle checkpoint [68]. Vinflunine, which fits in the family of *Vinca* alkaloids, halts the cell cycle at the G2/M checkpoint and is used against acute lymphoblastic leukemia and other lymphoid malignancies [19]. On the other hand, vincristine, derived from the leaves of the plant *Catharanthus roseus* (L.) G.Don. (formerly, *Vinca rosea*), has anti-microtubule effects on the cells of metastatic urothelial carcinoma [69] and breast cancer [70]. Nevertheless, there are reports of peripheral neuropathy associated with taxol [68]; neurotoxicity and few reports of blindness related to vincristine [71]; and anemia and neutropenia accompanying vinflunine [69]. Hence, in vivo and clinical testing for the newly emerging alkaloids cited above, like noscapine, liriodenine, isogravacridone, clausenidin, cycleanine, cryptolepine and brucine, which also induce cell cycle arrest, can equip us with novel anticancer drugs having reduced toxicity and more potent activities. Besides, these alkaloids can be tested for synergistic effects as they could represent hope for the marginal anticancer effects of taxol, vincristine and vinflunine.

#### 3.3.2. Alteration of the MAPK Pathway

In normal cells, the MAPK pathway plays a major role in linking extracellular signals to cellular responses and processes such as growth, proliferation, differentiation, migration and apoptosis [72]. Primarily, a certain ligand binds to a tyrosine receptor, activating the Ras protein through phosphorylation [73]. The latter then binds to effector proteins, such as B-Raf, which stimulate MEK 1 and 2. These kinases trigger ERK 1 and 2 activation, in turn activating several transcription factors of the AP-1 family. These agents eventually move to the nucleus and lead to the expression of genes encoding growth factors, cyclins, cytokines, and other important proteins involved in cell proliferation. Subsequently, the Ras-GTP-B-Raf complex is normally inactivated by GTPase-Activating Protein (GAP) shortly after its activation to avoid the overexpression of the cited proteins [61,74].

Numerous cancers have deregulations in the MAPK pathways that may lead to uncontrolled cellular divisions and consequently the occurrence of malignancies. Therefore, many anticancer drugs have been designed to modify and complement these disruptions. Two emerging alkaloids, rohitukine and hirsutine (Figure 1), activate p38 MAPKs leading to a dose-dependent cytotoxicity against breast cancer, ovarian, and lung cancer [41,43]. On the other hand, β-carboline alkaloid (Figure 3), isolated from *Peganum harmala* L., was found to exert its anticancer potential against human promyelocytic leukemia, prostate cancer, and gastric cancer by increasing the levels of Phosphatase and Tensin Homolog (PTEN) and decreasing the levels of ERK [45].

#### 3.3.3. Suppression of the NF-κB Pathway

Nuclear Factor-Kappa B (NF-κB) is an inducible nuclear transcription factor that activates genes involved in cell survival and proliferation. However, its down-regulation directs the cells towards apoptosis. The constitutive anomalous activation of this pathway leads to the up-regulation of proteins involved in cell invasion and angiogenesis especially those related to cell adhesion and survival [75,76]. Alkaloids have been found to suppress tumorigenesis by targeting the NF-κB pathway, namely by suppressing its activation and regulating its gene expression. Hirsutine, a bioactive alkaloid isolated from plants of the genus *Uncaria* exerts its cytotoxicity against murine breast cancer [44] and human breast cancer [43] cells by inhibiting the NF-κB pathway activation thereby abolishing cancer progression. In a similar fashion, α-tomatine, a tomato glycoalkaloid, induces apoptosis in human prostate cancer [49] and human lung adenocarcinoma cells [48] through the inhibition of NF-κB nuclear translocation in a dose-dependent manner. Taxol, in addition to inducing cell cycle arrest, acts through several other pathways such as p38 MAPK pathway and the NF-κB pathway [77]. Therefore, the success of this drug in the clinic could potentially indicate the future success of hirsutine, rohitukine, α-tomatine, and β-carboline alkaloid, all of which have been reported to target the NF-κB pathway.

### 3.4. Other Deadly Mechanisms: An Infinite Diversity

As presented throughout this review, many of the cited emerging alkaloids have been found to act through several molecular approaches. The alkaloids hirsutine and pretazettine as well as β-carboline alkaloids revealed additional modes of action, which will be presented below.

#### 3.4.1. Formation of G-Quadruplexes

β-carboline alkaloids lead to the formation of G-quadruplexes which consist of four guanine bases assembling in a three-dimensional square planar structure. These arrangements play regulatory roles on genes, most importantly on oncogenes, thus acting as antitumor agents against human promyelocytic leukemia, prostate cancer, and gastric cancer [46,78,79]. The formation of G-quadruplexes can be considered as a novel approach for cancer treatment and can have promising applications in anticancer drug design.

#### 3.4.2. HER2 Targeting

Anticancer drugs are most effective when they specifically target certain types of molecules. The isolation of the alkaloid hirsutine triggered the interest of many scholars, because it targets specifically the HER2 proteins activated in breast cancer cells. In fact, these proteins are encoded by the ERBB2 oncogene which is expressed in normal breast cells and which leads to the insertion of HER2 receptors. These molecules provide a system of control on the cell growth, division, and repair machineries. A mutation of ERBB2 ensues gene amplification resulting in the production of an enormous amount of HER2 receptors or an alteration causing the overexpression of the HER2 protein can also have the same result, eventually leading to loss of control in cell division and overgrowth. Such HER2-positive breast cancer cells grow faster, spread more quickly, and have a higher chance of relapse than usual breast cancer cells rendering them more treacherous and life-threatening [80]. Consequently, specialized anti-HER2 cancer agents, such as hirsutine, act by impeding the uncontrolled growth signals of cancer cells, thus causing their death. Two examples are the MDA-MB-453 and BT474 cell lines, which exhibited cytotoxic responses when exposed to the hirsutine and underwent apoptosis [43].

#### 3.4.3. Inhibition of the p-Glycoprotein ABCB1

Pretazettine (Figure 3) is a novel alkaloid which acts on several cancer cells in an interesting and uncommon molecular mechanism. In fact, this metabolite extracted from the flowering plants *Amaryllidaceae* battles breast cancer, cervical cancer, and skin epidermoid carcinoma by inhibiting p-glycoprotein (P-gp) which is also known as the ABCB1-member of the ABC proteins [47]. Multidrug resistance is a serious problem when treating cancer and this can occur through the up-regulation or activation of ATP-binding cassette (ABC) proteins which desensitize cancer cells to therapeutics. Hence, the inhibition of P-gp, one of the most important ABC proteins, increases the efficiency of cytotoxic agents which target the cancer cells [81]. Since pretazettine affects the p-glycoprotein ABCB1, it can be developed as a supportive agent to other cancer drugs, allowing them to overcome drug resistance.

## 4. Most Researched Alkaloids: *A Comprehensive Molecular Machinery*

Certain new alkaloids have been researched more than others and have been found to act strenuously on cancer cells, through a wide array of molecular modes of action making them worthy of being emphasized separately in this review.

### 4.1. Oxymatrine

**Figure 4:**
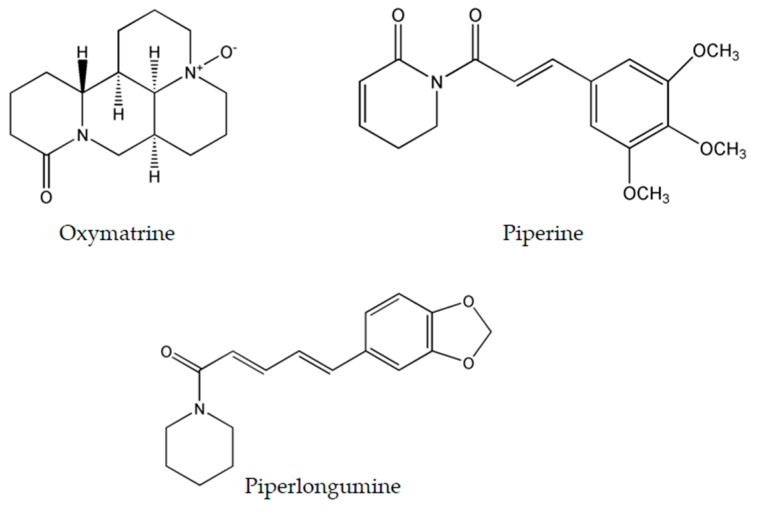
Chemical structure of most researched emerging plant-derived alkaloids oxymatrine, piperine and piperlongumine.

Oxymatrine (Figure 4), isolated from the shrubby *Sophora flavescens* Ait., has been studied thoroughly due to its diverse molecular effects [22]. Oxymatrine was found to induce time- and dose-dependent cytotoxicity in cells derived from breast [82], ovarian [83], prostate [84], colorectal [85], lung [86], gastric [87], cervical [88], and pancreatic cancers [89], as well as human hepatocellular carcinoma [90], laryngeal squamous cell carcinoma [91], osteosarcoma [92] and hemangioma [93]. These numerous targets are convoyed by an even wider range of molecular mechanisms induced by this alkaloid.

Oxymatrine has the potential to activate the intrinsic caspase pathway and induce apoptosis. The latter is coupled with the up-regulation of Bax and p53, the down-regulation of Bcl-2 [82,84,86,88,92,93], cell cycle arrest at the G0/G1 phase [91,93,94], and the inhibition of the NF-κB proliferative signaling pathway [85,89]. Moreover, oxymatrine reduces the expression of several other genes and pathways. For example, it is capable of inhibiting the epidermal growth factor receptor (EGFR) in gastric cancer cells which is involved in DNA synthesis and cell proliferation [87]. On the other hand, in cervical cancer cells, oxymatrine has the ability to constrain the activity of IMPDH2 enzyme [88] which is required for guanosine 5′-triphosphate (GTP) formation and thus DNA and RNA synthesis [95]. Oxymatrine exerts its anti-angiogenic effect in pancreatic cancer cells via targeting the NF-κB pathway and hindering the activity of the vascular endothelial growth factor (VEGF) involved in stimulating vasculogenesis and angiogenesis [89]. In addition, it suppresses the proliferation of gastric cancer cells by reducing the levels of phosphor-Cofilin (Ser3) and phosphor-LIMK1 (Thr508) whose inhibition leads to the halt of cancer cell migration and invasion [87]. Likewise, oxymatrine inhibits the Wnt/β-catenin signaling pathway in human breast cancer cells which is implied in the regulation of the cytoskeleton and the quantity of intracellular calcium [96]. Deregulation of the latter pathway causes disruptions in cell proliferation, migration and cell fate specification, thus favoring apoptosis of cancerous cells [75]. Oxymatrine is also capable of up-regulating miR-29b and down-regulating matrix metalloproteinase-2 (MMP-2) in cervical and laryngeal squamous cell carcinoma. miR-29b acts as a tumor suppressor and is generally inhibited in the majority of human cancers, whereas MMP-2 is involved in breaking down the extracellular matrix, and inhibiting the expression of the HPV16E7 gene which leads to increased levels of vimentin and fibronectin involved in cancer development [91,94].

Experiments on mice in which the in vivo anticancer activity of oxymatrine was evaluated revealed promising results. The intravenous injections of 40 and 80 mg/kg of oxymatrine, in human hepatocellular carcinoma derived mice models, resulted in dose-dependent inhibition of tumors by 39.4% and 63.7%, respectively [90]. The intraperitoneal treatment with 20 mg/kg of oxymatrine in osteosarcoma xenograft mice models significantly inhibited tumor growth by more than 50% with no associated cytotoxicity [92]. In pancreatic cancer xenograft tumors in nude mice, 100 mg/kg intraperitoneal injections of oxymatrine for three days a week had significant anti-proliferative and anti-angiogenic effects [89].

### 4.2. Piperine

Black pepper, *Piper nigrum* L. (piperaceae), is an extensively used spice worldwide. One of its major constituents is piperine (Figure 4) which belongs to the family of alkaloids. Although it is commonly known to exhibit immunomodulatory, anti-oxidant, anti-inflammatory, and anti-ulcer activity [97], piperine is an effective antineoplastic compound in vitro against a wide array of solid tumors such as colon [98], breast [99], osteosarcoma [100], prostate [101], lung [102], and rectal cancers [103]. Thorough examination proved that piperine is capable of arresting the cell cycle at different stages in many tumor cell lines. In colon cancer cells, piperine arrests cell cycle progression at the G1 phase with IC_50_ ranging between 53 and 126 μM. This arrest is commonly coupled with a decrease of cyclins D1 and D3 expression, reduced phosphorylation of Rb protein, and up-regulation of p21^WAF1^ and p27^KIP1^ expression [98]. Likewise, piperine induces G0/G1 cell cycle arrest in prostate cancer cells by up-regulating p21^WAF1^ and p27^Kip1^ expression and down-regulating cyclins D1 and A in a dose-dependent fashion [101]. However in osteosarcoma and lung cancer, piperine induces cell cycle arrest at the G2/M phase with IC_50_ ranging between 72 and 126 μM [100,102]. Recent studies have revealed the ability of piperine to increase ROS generation as means to induce apoptosis in rectal and colon cancer cells [98,103]. Moreover, in triple negative breast cancer cells MDA-MB-468, T-47D and MCF-7, it exerted its cytotoxicity via inducing mitochondrial-caspase-dependent apoptosis, reducing constitutive Akt activation and diminishing the expression of MMP-2 protein [99]. Nonetheless, in HER2-overexpressing breast cancer cells, piperine inhibited the activation of AP-1 and NF-κB pathway by interfering with Akt, p38 MAPK and ERK1/2 proliferative pathways resulting in caspase-3-activated apoptosis [104].

A single animal study has been conducted to measure the anti-neoplastic efficacy of piperine. Immuno-compromised female NOD-SCID mice were injected with triple-negative breast cancer cells in the upper left mammary fat pad. The intratumoral injections of piperine at 0.2 mg/kg led to 54% reduction of tumor volume in comparison to the control with insignificant associated cytotoxicity [99].

### 4.3. Piperlongumine

Piperlongumine (Figure 4), a natural bioactive molecule isolated from the long pepper (*Piper longum* L.) plant, is an efficacious alkaloid that have shown to be promising in having anticancer activity [105] by selectively inducing ROS generation and apoptosis in solid and liquid tumors including gastric [106], glioma [107], breast [108], activated B-cell lymphoma [109], renal [110], prostate [111], and colon cancers [112]. At the molecular level, piperlongumine was found to raise the intracellular levels of ROS in gastric cancer cells with IC_50_ value ranging between 2.3 and 6.0 μM. The up-regulation of ROS was coupled to the inhibition of the antioxidant enzyme TrxR1, the decreased expression levels of Bcl-2, the decreased expression of telomerase reverse transcriptase gene, thus resulting in cell cycle arrest at the G2/M phase [106,113]. Piperlongumine enhanced cisplatin antitumor ability in a synergetic manner against head-and-neck cancer by targeting stress responses, thereby increasing the intracellular levels of ROS and inducing cell death [114]. Additionally, treatment of multiple high-grade glioma spheres by piperlongumine caused increased ROS levels and increased oxidative inactivation of peroxiredoxin 4, thus inducing endoplasmic reticulum stress and apoptosis [107]. In addition to inducing ROS generation, piperlongumine inhibited the NF-κB pathway by either hindering TNF-α in activated B cell lymphoma [109], or down-regulating c-Met expression and its downstream signaling pathways such as Erk/MAK and STAT3 pathways in renal cell carcinoma [110]. In breast and colon cancer, piperlongumine inhibited the proliferative STAT3 pathway and activated ERK signaling circuitry respectively as a means of inducing cell death [108,112].

Several animal studies have been conducted to explore the potential in vivo therapeutic efficacy of piperlongumine. In gastric xenograft mice models, a dose of 12 mg/kg of piperlongumine resulted in significantly smaller tumors without any associated weight loss [113]. On the other hand, intraperitoneal injection of 2.5 mg/kg piperlongumine in head and neck xenograft models caused significant decrease of the growth rate of the tumors. In the same study, combining piperlongumine with 5 mg/kg of cisplatin revealed a possible synergistic effect, as the administration of both drugs gave more pronounced decrease in tumor growth in comparison to single treatments [114]. In nude mice models bearing human gastric cell tumors, the intraperitoneal injections of 3.6 mg/kg, once per day, led to remarkably reduced tumor volumes, and was coupled with a decrease in Ki67 expression and enhanced GADD45α expression with no apparent toxicity [106]. Moreover, a 20 mg/kg dose of piperlongumine, used three times a week against renal xenograft mice models was tolerated by all animals as the tumor volumes decreased by 50% with no recorded signs of toxicity [110]. Standard chemotherapy and its sub-type, targeted therapy, rely on one or few molecular modes of action to treat cancer [115]. In fact, a combination of chemotherapy and targeted therapy is being increasingly used in cancer therapy, especially in cancers with developed chemo-resistance [67]. As summarized in Table 4, oxymatrine, piperine, and piperlongumine, have the capacity to interfere with various molecules involved in cancer proliferation and survival pathways, thus qualifying to serve as new multi-potent drugs to be considered for clinical trials.

## 5. Selectivity against Cancer Cells: *The Trail to Non-Toxic Anticancer Agents*

The effectiveness of newly formulated anticancer drugs resides in their ability to distinguish between the hyper-proliferative cancer cells and the normally growing ones [67]. Although there have been many advances in cancer therapeutics, few clinically used agents have been identified to selectively target cancer cells without exerting cytotoxic effects on the neighboring normal tissues [117]. Remarkably, many of the newly emerging alkaloids have shown to specifically reduce the in vitro viability of cancer cells with minimal effects on normal cell lines. For instance, treatment of healthy human prostate cells, specifically the PNT1B cell line, with oxymatrine resulted in an unaffected cell viability curve even after prolonged exposure (72 h) at 8 mg/mL [84]. On the other hand, piperlongumine has been tested on several normal cells, such as normal gastric epithelial GES-1 cells [113], astrocytes [107], normal GCB-DLBCL [109], non-tumorigenic breast epithelial MCF-10A and MCF-12A cells [108], and normal colon NCM 460 cell lines [112]. Strikingly, it had minimal effect on these cells even after high exposure periods with concentrations ranging between 5 and 10 μM. In a similar fashion, liriodenine showed no evidence of toxicity on normal ovarian WRL-68 cells at concentrations higher than 10 μM [25], whereas the inhibition of normal ovarian surface epithelial OSE cell viability by cycleanine was almost ten folds lower in comparison to ovarian cancer cells [34]. In addition, cathachunine treatment had much lower inhibitory effects on human umbilical vein cells, a well-used model of normal human endothelial cells. In fact, Hoechst staining of the latter cells indicated an absence of apoptosis upon treatment thereby confirming the selectivity of cathachunine against human leukemic cells [35]. Last but not least, the treatment of normal human prostate RWPE-1 cell line with 30 μM of subditine [39] or 5 μM of α-tomatine [49] showed insignificant inhibitory effects asserting their specificity and selectivity against cancer cells.

## 6. Future Perspectives

In the pursuit of finding the utmost effective anticancer therapies, many plant-derived alkaloids have been studied intensely then developed into FDA-approved anticancer drugs. The dilemma is that while these drugs such as Taxol [68], camptothecin [118], topotecan [16], and the *Vinca* alkaloids vincristine and vinflunine [118], have all been approved for the treatment of various types of solid and liquid tumors, they exhibit a certain level of recorded cytotoxic reactions in patients such as neurotoxicity [57] and abdominal problems [77]. The resurgence of plant-derived alkaloid anticancer agents, as manifested by clinical trials and FDA-approved drugs, have urged the search for novel anti-neoplastic alkaloids. In this review, we aimed to highlight 20 new different plant-derived alkaloids which are relatively not highly studied, yet have shown to be effective against numerous cancer types with promising IC_50_ values. We have attempted to group these alkaloids by their molecular modes of action, and have exposed their vast intracellular and extracellular effects, and underlined their potency as therapeutic agents. These new plant-based drug candidates act through various modes of action; some cause DNA damage, modulate the PI3k/Akt signal transduction cascade, and elevate the levels of ROS, whereas others induce a caspase or a MAPK pathway response in cells. However, many act by arresting the cell cycle at various checkpoints and down-regulating the NF-κB survival pathway. Few lead to the formation of G-quadruplexes while others act as inhibitors of the p-glycoprotein ABCB1 thus serving as agents against drug resistance in cancer cells. These molecular approaches represent the basis for more advanced research on these alkaloids.

Importantly, similar to herbivorous predators, human beings can be vulnerable to the toxicity of plant-derived alkaloids. The future challenge would be to make use of the chemical diversity of the newly emerging alkaloids in order to explore their cytotoxic selectivity to a multitude of cancer cells and to identify potential targets for improved treatment strategies in cancer patients. In this context of improved selectivity against cancer, plant-derived alkaloids are being continuously tested on normal cells and tissues to evaluate their specificity. Oxymatrine [84], piperlongumine [107,108,109,112,113], liriodenine [25], cycleanine [34], cathachunine [35], subditine [39], and α-tomatine [49] have been found to exert insignificant cytotoxic effects on normal cells even when exposed for prolonged periods at elevated concentrations. Such interesting results warrant the in vivo testing of these compounds as this could yield to promising outcomes. If their anticancer efficacy and lack of toxicity is documented in vivo, these alkaloids would constitute promising potential therapeutic agents worthy of clinical translation. Of the 20 alkaloids presented in this review, three have been well researched against different types of cancer and have been found to be effective both in vitro and in vivo, namely oxymatrine, piperine and piperlongumine. We have presented data that these three drugs specifically target different types of cancer cells and manifest specific mechanisms of action in accordance with the type of cancer they act on (demonstrated in Table 4). Although only a small number of in vivo studies have been conducted on piperine, piperlongumine, and oxymatrine in mice xenograft models, the studies performed so far have yielded significant and interesting results with almost negligible associated induced toxicity on normal cells, thus making these compounds suitable for further development.

Coming to an understanding of how specifically these three alkaloids function systematically in addition to the rest of the discussed alkaloid molecules will definitely help researchers create novel combinations of therapies which supposedly will have higher efficacy and lower cytotoxicity on normal tissues. As such, further research is highly essential to explore additional mechanisms of action of these emerging alkaloids and to study their effects on other types of cancers. Future studies in molecular chemistry and molecular docking analysis are mandatory to gain an insight on how these prodigious molecules interact with the cellular components. This would pave the way for designing new anticancer drugs of semi-synthetic origin and provide the basis for combining natural compounds with mainstream chemotherapy and targeted therapy for the purpose of enhancing their antineoplastic abilities and reducing their unwanted cytotoxicity in patients.

## Figures and Tables

**Table 1:** Activity of newly emerging anti-neoplastic plant-derived alkaloids on cancer cell lines.

Alkaloids	Plant Source	Type of Cancer	Cell Lines (IC_50_)	References
Liriodenine	*Enicosanthellum pulchrum* (King) Heusden	Ovarian	CAOV-3 (37.3 μM)	[25,26]
			SKOV-3 (68.0 μM)	
		laryngocarcinoma	HEp-2 (2.332 μM)	
Noscapine	*Papaver somniferum* L.	glioma cell cancer	LN229 (70 μM)	[27,28]
			A172 (20 μM)	
			U251 (40 μM)	
		neuroblastoma	SK-SY5Y, SH-EP1, SK-N-MC, SK-N-AS, LA1-55N, NB1643, NB1691, SK-N-SH, and IMR32 (IC_50_ range for all cell lines: from 21 to 100 μM)	[27,28]
		cervical	HeL	
			Ca Ski	
		colon	Caco-2	
			T84	
		ovarian	SK-OV-3	
			and SigC	
		prostate	DU145	
		human lymphoblast	CEM (14.5 μM)	
		human cervix	HeLa (24.0 μM)	
		lung adenocarcinoma	A549 (72.9 μM)	
		breast epithelial	MCF-7 (42.3 μM)	
		breast	MDA-MB-231 (20.15 µM)	
			MCF-7 (15.47 µM)	
Cryptolepine	*Cryptolepis sanguinolenta* *Sida acuta* Brum.f. *Sida cordifolia* L.	lung adenocarcinoma	A549	[29,30,31]
		Osteosarcoma	MG63	
		T-cell leukemia	CCRF-CEM	
			CEM/VM-1	
		multiple myeloma	RPMI 8226-S	
			8226/Dox	
			8226/LR5	
		histiocytic lymphoma	U-937-GTB	
			U-937/Vcr	
		small cell lung cancer	NCI-H69	
			H69/AR	
		renal adenocarcinoma	ACHN	
		cervical adenocarcinoma	HeLa	
		immortalized normal retinal epithelial cells	hTERT-RPE (mean IC_50_ of all cell lines: 0.9 μM)	
Clausenidin	*Clausena excavata* Burum.f.	colon	HT-29 (13.8 μg/mL)	[32]
Isogravacridone chlorine	*Ruta graveolens* L.	breasts	MDA-MB-231 (2.27 μM)	[33]
Cycleanine	*Triclisia subcordata* Oliv.	ovarian	Ovcar-8 (10 μM)	[34]
			A2780 (7.6 μM)	
			Ovcar-4 (7.2 μM)	
			Igrov-1 (14 μM)	
Cathachunine	*Catharanthus roseus* (L.) G.Don.	leukemia	HL60 (9.1 μM)	[35]
			K562 (9.3) μM	
Brucine	*Strychnos nux-vomica* L.	lung	PC-9	[36,37,38]
		hepatocellular carcinoma	HepG2	
			SMMC-7721	
		colon	LoVo (15.1 μM)	
		lung	PC-9	
Subditine	*Nauclea subdita* (Korth.) Steud.	prostate	LNCaP (12.24 µM)	[39]
			PC-3 (13.97 µM)	
Scutebarbatine-A (SBT-A)	*Scutellaria barbata* D.Don.	lung	A549 (39.21 μg/mL)	[40]
Rohitukine	*Dysoxylum binectariferum* Hook.f.	breast	T47D (50 µM), and	[41]
			MIDAMB273 (3 µM)	
			MCF7 (15 µM)	
		ovarian	SKOV3 (20 µM)	
		lung	A549 (40 µM)	
Tabernaelegantine C	*Tabernaemontana elegans* Stapf	colon	HCT116 (20 µM)	[42]
	*Muntafara sessilifolia* Baker			
Tabernaelegantinine B	*Tabernaemontana elegans* Stapf	colon	HCT116 (20 µM)	[42]
	*Muntafara sessilifolia* Baker		MRC-5 (0.47 µM)	
Hirsutine	Plants of genus *Uncaria*	human breast	MDA-MB-453	[43,44]
		mouse mammary carcinoma	BT474	
			4T1	
β-carboline	*Peganum harmala* L.	human promyelocytic leukemia	HL-60 (3.48 μg/mL)	[45,46]
		prostate	PC-3 (10.59 μg/mL)	
		gastric	SGC-7901 (11.53 μg/mL)	
Pretazettine	*Amaryllidaceae* (genus *Amaryllis* L.)	breast	MCF7 (7.869 µM)	[47]
		cervical	HeLa (8.853 µM)	
		skin epidermoid carcinoma	A431 (5.373 µM)	
α-tomatine	*Lycopersicon esculentum* Mill.	human lung adenocarcinoma	A549 cells	[48,49]
		human prostatic adenocarcinoma	PC-3 Cells (1.67 µM)	

**Table 2:** Emerging DNA damaging plant-derived alkaloids.

Alkaloid	Type of Cancers It Protects against	Exact Pathway	References
Cathachunine	leukemia	↑ROS levels	[35]
Subditine	prostate	↑ROS levels	[39]
Rohitukine	breast, ovarian, lung	↑ROS levels	[41]
Hirsutine	human, breast, cancer, mouse mammary carcinoma	Damaging DNA	[43,44]
		↑γH2AX	
		Suppression of Akt Pathways	

**Table 3:** Apoptotic mechanisms of emerging plant-derived alkaloids.

Alkaloid	Mechanisms of Action	References
Liriodenine	Cleavage of caspases-3 and -9	[25]
	Efflux of cytochrome c	
	↑Bax, ↑p53 expression, ↓Bcl-2 and ↓survivin	
Cryptolepine	↑p53 and p21^Cip1/WAF1^	[29,31]
Clausenidin	Cleavage of caspases-3 and -9	[32]
	Efflux of cytochrome c	
	↑Bax and ↑Apaf-1	
Isogravacridone chlorine	Cleavage of caspase-9	[33]
Cathachunine	Cleavage of caspases-3, -9 and PARP	[35]
	Disruption of mitochondrial membrane potential	
	Efflux of cytochrome c	
	activation of caspases-3 and -9	
	↑Bax and ↓Bcl-2	
Brucine	↑Bax and ↓Bcl-2 expression	[37]
Subditine	Cleavage of caspases-3 and -9	[40]
	Efflux of cytochrome c	
	↑Bax, ↑p53 expression, ↓Bcl-2, and ↓Bcl-x	
Scutebarbatine A (SBT-A)	Cleavage of caspases-3 and -9	[40]
	Efflux of cytochrome c	
	↑Bax and ↓Bcl-2	
Rohitukine	Cleavage of caspases-3 and -9	[41]
	Efflux of cytochrome c	
	↓Bcl-2	
Tabernaelegantinine B	Cleavage of caspases-3 and -8	[42]
Tabernaelegantine C		

**Table 4:** Activities and mechanisms of action of most researched emerging plant-derived alkaloids on cancer cell lines.

Alkaloid	Plant Source	Type of Cancer	Cell Lines (IC_50_/ED_50_)	Mechanism of Action	References
Oxymatrine	*Sophora flavescens* Ait.	breast	MCF7	↑Bax and ↓Bcl-2	[82]
		ovarian	OVCAR-3	Cleavage of caspase-3, ↑miR-29b and ↓matrix metalloproteinase-2 (MMP2)	[83]
		prostate	DU145, PC-3	↑Bax, ↑p53, and ↓Bcl-2	[84]
		colorectal	RKO HCT116 SW480	Regulation of EMT markers (↑E-cadherin, ↓Snail and ↓N-cadherin) Inhibition of NF-κB activation, ↓p65	[85]
		lung	A549	↑Bax and ↓Bcl-2	[86]
		gastric	MKN-45 BGC823 SGC7901 HEK293	G1 cell cycle arrest Disruption of mitochondrial membrane potential Inhibition of EGFR (p-Tyr845) ↓CyclinD1, ↓CDK4/6 ↑Bax and ↓Bcl-2 ↑ caspases-3 and -9 mRNA level ↓phospho-Cofilin (Ser3), phospho-LIMK1 (Thr508) levels, and ↓MMP2	[87]
		cervical	CaSki	G0/G1 and S cell cycle arrest ↓HPV16E7	[94]
		cervical	HeLa	↓IMPDH2 ↓intracellular GTP	[88]
		human hepatoma carcinoma	Hep-G2 (1.32 mg/mL) SMMC-7721 (1.21 mg/mL)	↑Bax and ↓Bcl-2 and ↑caspase-3 mRNA level	[90]
		laryngeal squamous cell carcinoma	Hep-2 (7 mg/mL)	G0/G1 cell cycle arrest ↓HPV16E7 gene	[91]
		Pancreatic	PANC-1 (1 mg/mL)	Inhibition of NF-κB activity, ↓VEGF	[89]
		osteosarcoma	MNNG/HOS (72.50 μg/mL)	↑Bax and ↓Bcl-2 Disruption of mitochondrial membrane potential Efflux of cytochrome c Cleavage of caspases-3 and -9 Inactivation of PI3K/Akt pathway ↑Bax and ↓Bcl-2	[92]
		osteosarcoma	MG-63 (0.75 mg/mL)	Disruption of mitochondrial membrane potential Cleavage of caspases-3 and -9 ↑Bax and ↓Bcl-2	[116]
		hemangioma	HDEC	↓HIF-1ɑ, ↓VEGF, ↑Bax and ↑p53, and ↓Bcl-2 G0/G1 cell cycle arrest and ↓cyclinD1	[93]
		breast	MCF-7	↓SP and ↓Wnt/β-catenin signaling pathways	[96]
Piperine	*Piper nigrum* L. *Piper longum* L.	colon	CaCo-2 (54 μM) SW480 (126 μM) HCT116 (118 μM) HT-29 (53 μM)	G1 cell cycle arrest Disruption of mitochondrial membrane potential Cleavage of caspases-3, -9 and PARP ↑ROS Induction of endoplasmic reticulum stress	[98]
		triple-negative breast cancer	MDA-MB-468 T-47D MCF-7	Disruption of mitochondrial membrane potential Efflux of cytochrome c G1/S and G2/M cell cycle arrest Induction of caspase-dependent apoptosis ↓p-Akt, ↑p21^Waf1/Cip1^ ↓MMP-2/-9 mRNA levels	[99]
		HER2-overexpressing breast cancer	SKBR3 (50 μM) BT-474 (50 μM) MCF-7 (200 μM) MDA-MB-231	Cleavage of caspase-3 and PARP ↓SREBP-1 and ↓FAS mRNA levels ↓HER2 Inhibition of Akt, MAPK, AP-1 and NF-κB activation Suppression of migration	[104]
		osteosarcoma	HOS (72 μM) U2OS (126 μM)	G2/M cell cycle arrest ↓cyclinB1, ↑p-CDK1, ↑p-Chk2 Inhibition of p-Akt Activation of c-JNK, p38MAPK- ↑TIMP-1/-2 and ↓MMP-2/-9	[100]
		prostate	DU145 (74.4 μM) PC-3 (226.6 μM) LNCaP (111 μM)	G0/G1 cell cycle arrest Cleavage of caspase-3 Induction of autophagy *via* ↑LC3B-II and formation of LCb3 puncta ↑p21^Cip1^, ↑p27^Kip1^, ↓cyclin D1, ↓cyclin A	[101]
		lung	A549 (122 μg/mL)	G2/M phase cell cycle arrest Cleavage of caspases-3 and -9 ↑Bax, ↑p53, and ↓Bcl-2	[102]
		rectal	HRT-18	G0/G1cell cycle arrest ↑ROS	[103]
Piperlon-gumine	*Piper longum* L.	gastric	SGC-7901 (2.3 μM) BGC-823 (3.9 μM) KATO III: (6.0 μM)	G2/M cell cycle arrest ↓MDM-2, ↓Cyclin B1, and ↓Cdc2 ↑ROS and ↓TrxR1 Cleavage of caspase-3 ↑Bax and ↓Bcl-2 Induction of ROS-dependent endoplasmic reticulum stress Induction of ROS-dependent mitochondrial dysfunction	[113]
		head and neck	AMC-HN SNU HN30 HN31	↑ROS ↑p53, ↑PUMA, ↑p-JNK, ↓GSTP1 and ↑p21^Waf1/Cip1^ Cleavage of PARP	[114]
		glioma	HGG 1123 HGG MD13	↑ROS levels ↓PRDX4	[107]
		large B cell lymphoma ABC-DLBCL	OCI-Ly10 U2932 DB	Inhibition of TNF-α and p65 nuclear import ↓NF-κB activity ↓survivin, Bcl-2, ↑Bax, nd ↑p21 Cleavage of caspases-3 and -9	[109]
		breast myeloid leukemia	MCF7 (0.9 μM) MDA-MB-453 (0.9 μM) T-47D (2.7 μM), Kasumi-1 (3.7 μM)	↓ p-STAT3 Inhibition of STAT3 binding to its immobilized phosphopeptide ligand Cleavage of caspase-3 ↑p53, ↓survivin, ↓Bcl-2, ↓Bcl-x, ↓XIAP, and ↓CIAP mRNA levels	[108]
		gastric	AGS HGC27	↑ROS Cleavage of caspases-3, -7, -9 and PARP G2/M cell cycle arrest and ↑GADD45ɑ ↓CyclinB1, ↓cdc2, ↓XIAP, and ↑p21 ↓telomerase reverse transcriptase gene Induction of endoplasmic reticulum stress	[106]
		renal carcinoma	786-O PNX0010 (ED_50_: 1.6, 2.3 μM respectively)	↓c-Met ↑ROS Inhibition of Erk/MAPK, STAT3, Akt/mTOR and NF-κB pathways	[110]
		prostate	PC-3 DU-145 (ED_50_: 4.9, 3.4 μM respectively)	Inhibition of TNF-α and p65 nuclear import Inhibition of NF-κB pathway ↓Il-6, ↓IL-8, ↓MMP-9 Inhibition surface expression of ICAM-1	[111]
		colon	HT-29 (10.1 μM) HCT 116 (6.4 μM)	Cleavage of caspase-3 Induction of ERK signaling pathway *via* ↑p-ERK	[112]
